# Tris(pyridin-2-yl­methanol)nickel(II) hexa­fluoridophosphate trifluoro­acetate

**DOI:** 10.1107/S160053681104431X

**Published:** 2011-10-29

**Authors:** Tomohiko Hamaguchi, Tomoko Nagata, Satoshi Kawata, Isao Ando

**Affiliations:** a Department of Chemistry, Faculty of Science, Fukuoka University, 8-19-1 Nanakuma, Jonan-ku, Fukuoka 814-0180, Japan

## Abstract

In the crystal structure of the title complex, [Ni(C_6_H_7_NO)_3_](PF_6_)(C_2_F_3_O_2_), the Ni^II^ ion is in a slightly distorted octa­hedral NiO_3_N_3_ coordination geometry with each of the three N and three O atoms in a meridional coordination. In the crystal, the complex mol­ecules and the trifluoro­acetate anions are connected *via* O—H⋯O hydrogen bonding into layers parallel to the *ab* plane.

## Related literature

For related complexes, see: Ito & Onaka (2004 ▶); Kermagoret & Braunstein (2008 ▶).
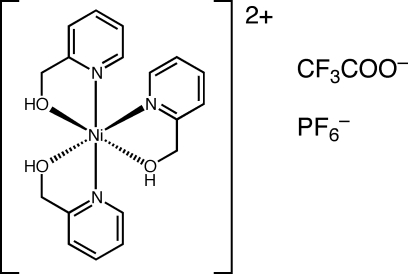


## Experimental

### 

#### Crystal data

[Ni(C_6_H_7_NO)_3_](PF_6_)(C_2_F_3_O_2_)
*M* *_r_* = 644.08
Triclinic, 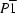

*a* = 9.6381 (2) Å
*b* = 11.9668 (4) Å
*c* = 11.9892 (3) Å
α = 109.950 (1)°
β = 95.348 (1)°
γ = 101.411 (1)°
*V* = 1254.60 (6) Å^3^
*Z* = 2
Mo *K*α radiation
μ = 0.94 mm^−1^
*T* = 200 K
0.40 × 0.30 × 0.20 mm

#### Data collection

Rigaku R-AXIS RAPID diffractometer
Absorption correction: multi-scan (*ABSCOR*; Rigaku, 1995 ▶) *T*_min_ = 0.813, *T*_max_ = 1.000
12517 measured reflections
5736 independent reflections
5234 reflections with *I* > 2σ(*I*)
*R*_int_ = 0.018

#### Refinement

*R*[*F* ^2^ > 2σ(*F* ^2^)] = 0.033
*wR*(*F* ^2^) = 0.088
*S* = 1.05
5736 reflections
376 parameters
9 restraints
H-atom parameters constrained
Δρ_max_ = 0.50 e Å^−3^
Δρ_min_ = −0.45 e Å^−3^

### 

Data collection: *RAPID-AUTO* (Rigaku, 2002 ▶); cell refinement: *RAPID-AUTO*; data reduction: *RAPID-AUTO*; program(s) used to solve structure: *SIR2004* (Burla *et al.*, 2005 ▶); program(s) used to refine structure: *SHELXL97* (Sheldrick, 2008 ▶); molecular graphics: *Yadokari-XG* (Wakita, 2001 ▶; Kabuto *et al.*, 2009 ▶), *ORTEP-3* for Windows (Farrugia, 1997 ▶) and *Mercury* (Macrae *et al.*, 2008 ▶); software used to prepare material for publication: *Yadokari-XG* and *publCIF* (Westrip, 2010 ▶).

## Supplementary Material

Crystal structure: contains datablock(s) I, global. DOI: 10.1107/S160053681104431X/nc2249sup1.cif

Structure factors: contains datablock(s) I. DOI: 10.1107/S160053681104431X/nc2249Isup2.hkl

Additional supplementary materials:  crystallographic information; 3D view; checkCIF report

## Figures and Tables

**Table:** 

Ni1—O1	2.0461 (12)
Ni1—N2	2.0601 (14)
Ni1—O2	2.0647 (12)
Ni1—N1	2.0662 (14)
Ni1—O3	2.0714 (12)
Ni1—N3	2.0769 (14)

**Table:** 

O1—Ni1—N1	78.11 (5)
N2—Ni1—O2	78.53 (5)
O3—Ni1—N3	78.09 (5)

**Table 2:** Hydrogen-bond geometry (Å, °)

*D*—H⋯*A*	*D*—H	H⋯*A*	*D*⋯*A*	*D*—H⋯*A*
O1—H1⋯O4^i^	0.87	1.76	2.6003 (19)	162.5
O2—H2⋯O5^ii^	0.92	1.77	2.6965 (18)	175.8
O3—H3⋯O5^iii^	0.98	1.65	2.6267 (18)	173.8

Symmetry codes: (i) 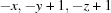; (ii) 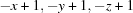; (iii) 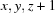.

**Fig. 1.**
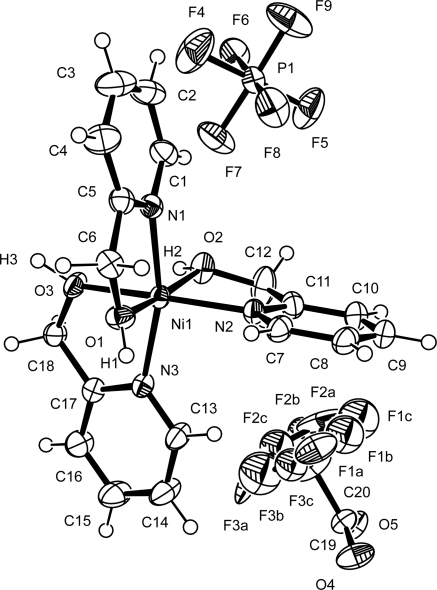
ORTEP drawing for the title complex with labeling showing 50% probability displacement ellipsoids. Please note: The trifluoroacetate anion is disordered.

**Fig. 2.**
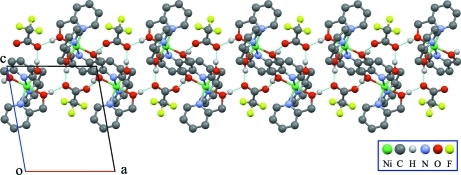
Crystal structure of the title compound view along the b-axis. The C-H H atoms, the PF6 anions and the disordered F atoms with lower occupation of the trifluoroacetate anions are omitted for clarity. O—H···O hydrogen bonding is shown as dashed blue lines.

**Fig. 3.**
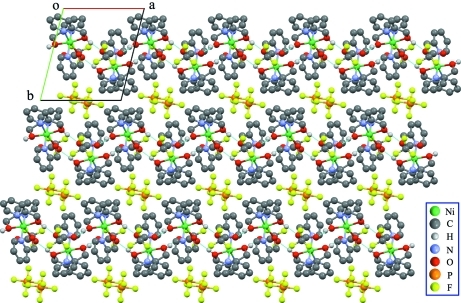
Crystal structure of the title compound with view along the c-axis. The C-H H atoms and the disordered F atoms with lower occupation of the trifluoroacetate anions are omitted for clarity.

## supplementary crystallographic information

### Comment

The crystal structure of the title compound is composed of [Ni^II^(C_6_H_7_ON)_3_]^2+^ cations, hexafluorophosphate and trifluoroacetate anions. The Ni^II^ ion is in a slightly distorted octahedral coordination, comprising three N atoms and three O atoms from three pyridine-2-methanol ligands (Fig. 1 and Table 1). The three N and three O atoms make a meridional NiO_3_N_3_ coordination and the mean bite angle of the pyridine-2-methanol ligand amount to 78.2 (2) °.

In the crystal structure the complexes are connected via O—H···O hydrogen bonding between the hydroxy H atoms of the pyridine-2-methanol ligand and the O atoms of the trifluoroacetate anion into layers that are parallel to the a/b plane. (Fig. 2 and 3 and Table 2).

### Experimental

A solution of NiSO_4_.6H_2_O (0.5 mmol) in H_2_O (1 ml) was added to the solution of pyridine-2-methanol (1.5 mmol) in H_2_O (3 ml). Afterwards NH_4_PF_6_ (6.0 mmol) and CF_3_COONa (2.5 mmol) were added to the resulting blue solution. The resulting pale blue precipitate was collected. The crude product was purified by recrystallization from acetone and water. The blue prism-like crystals were obtained a few days later on slow evaporation of the solvent.

### Refinement

The O–H H atoms were located in a difference Fourier map and the coordinates were fixed. Their *U*_iso_(H) values were set to 1.5*U*_eq_(O). Other H atoms were placed at calculated positions and were treated as riding on the parent C atoms, with C–H = 0.93 (CH) and 0.97 (CH_2_) Å and with *U*_iso_(H) = 1.2*U*_eq_(C). Three F atoms in CF_3_COO anions are rotationally disordered between three positions. The two parts of lower occupation were refined only isotropic (sof. 0.6:0.24:0.16).

### Crystal data

**Table:**

[Ni(C_6_H_7_NO)_3_](PF_6_)(C_2_F_3_O_2_)	*V* = 1254.60 (6) Å^3^
*M**_r_* = 644.08	*Z* = 2
Triclinic, *P*1	*F*(000) = 652
Hall symbol: -P 1	*D*_x_ = 1.705 Mg m^−^^3^
*a* = 9.6381 (2) Å	Mo *K*α radiation, λ = 0.71075 Å
*b* = 11.9668 (4) Å	µ = 0.94 mm^−^^1^
*c* = 11.9892 (3) Å	*T* = 200 K
α = 109.950 (1)°	Block, blue
β = 95.348 (1)°	0.40 × 0.30 × 0.20 mm
γ = 101.411 (1)°	

### Data collection

**Table:**

Rigaku R-AXIS RAPID diffractometer	5234 reflections with *I* > 2σ(*I*)
graphite	*R*_int_ = 0.018
ω scans	θ_max_ = 27.5°, θ_min_ = 3.1°
Absorption correction: multi-scan (*ABSCOR*; Rigaku, 1995)	*h* = −12→11
*T*_min_ = 0.813, *T*_max_ = 1.000	*k* = −15→15
12517 measured reflections	*l* = −15→15
5736 independent reflections	

### Refinement

**Table:**

Refinement on *F*^2^	Primary atom site location: structure-invariant direct methods
Least-squares matrix: full	Secondary atom site location: difference Fourier map
*R*[*F*^2^ > 2σ(*F*^2^)] = 0.033	Hydrogen site location: inferred from neighbouring sites
*wR*(*F*^2^) = 0.088	H-atom parameters constrained
*S* = 1.05	*w* = 1/[σ^2^(*F*_o_^2^) + (0.0486*P*)^2^ + 0.5841*P*] where *P* = (*F*_o_^2^ + 2*F*_c_^2^)/3
5736 reflections	(Δ/σ)_max_ = 0.001
376 parameters	Δρ_max_ = 0.50 e Å^−^^3^
9 restraints	Δρ_min_ = −0.45 e Å^−^^3^

### Special details

**Table:**

Geometry. All esds (except the esd in the dihedral angle between two l.s. planes) are estimated using the full covariance matrix. The cell esds are taken into account individually in the estimation of esds in distances, angles and torsion angles; correlations between esds in cell parameters are only used when they are defined by crystal symmetry. An approximate (isotropic) treatment of cell esds is used for estimating esds involving l.s. planes.
Refinement. Refinement of *F*^2^ against ALL reflections. The weighted *R*-factor *wR* and goodness of fit *S* are based on *F*^2^, conventional *R*-factors *R* are based on *F*, with *F* set to zero for negative *F*^2^. The threshold expression of *F*^2^ > σ(*F*^2^) is used only for calculating *R*-factors(gt) *etc*. and is not relevant to the choice of reflections for refinement. *R*-factors based on *F*^2^ are statistically about twice as large as those based on *F*, and *R*- factors based on ALL data will be even larger.

### Fractional atomic coordinates and isotropic or equivalent isotropic displacement parameters (Å^2^)

**Table:**

	*x*	*y*	*z*	*U*_iso_*/*U*_eq_	Occ. (<1)
Ni1	0.18393 (2)	0.369025 (18)	0.808996 (17)	0.02212 (7)	
O1	0.00845 (13)	0.39356 (12)	0.88953 (12)	0.0304 (3)	
H1	−0.0596	0.4198	0.8609	0.046*	
O2	0.35252 (13)	0.32008 (12)	0.72559 (12)	0.0337 (3)	
H2	0.4425	0.3733	0.7547	0.051*	
O3	0.33165 (14)	0.49798 (11)	0.95482 (11)	0.0316 (3)	
H3	0.3431	0.5104	1.0408	0.047*	
N1	0.16982 (15)	0.24566 (12)	0.89561 (13)	0.0257 (3)	
N2	0.07694 (15)	0.24685 (13)	0.64160 (13)	0.0264 (3)	
N3	0.20297 (15)	0.52595 (13)	0.76794 (12)	0.0255 (3)	
C1	0.26677 (19)	0.17914 (16)	0.90061 (18)	0.0338 (4)	
H4	0.3381	0.1775	0.8509	0.041*	
C2	0.2656 (2)	0.11372 (19)	0.9755 (2)	0.0447 (5)	
H5	0.3343	0.0670	0.9765	0.054*	
C3	0.1636 (3)	0.1169 (2)	1.0488 (2)	0.0530 (6)	
H6	0.1624	0.0740	1.1025	0.064*	
C4	0.0627 (2)	0.1836 (2)	1.0432 (2)	0.0458 (5)	
H7	−0.0093	0.1864	1.0924	0.055*	
C5	0.06813 (18)	0.24611 (15)	0.96467 (16)	0.0286 (3)	
C6	−0.04137 (18)	0.31692 (16)	0.95294 (17)	0.0297 (4)	
H8	−0.1347	0.2596	0.9086	0.036*	
H9	−0.0553	0.3674	1.0338	0.036*	
C7	−0.06644 (19)	0.20268 (16)	0.60926 (16)	0.0306 (4)	
H10	−0.1254	0.2263	0.6681	0.037*	
C8	−0.1309 (2)	0.12438 (17)	0.49383 (18)	0.0377 (4)	
H11	−0.2324	0.0945	0.4734	0.045*	
C9	−0.0448 (2)	0.09037 (18)	0.40852 (18)	0.0418 (5)	
H12	−0.0866	0.0372	0.3282	0.050*	
C10	0.1021 (2)	0.13424 (19)	0.44087 (17)	0.0400 (4)	
H13	0.1628	0.1117	0.3833	0.048*	
C11	0.1602 (2)	0.21193 (17)	0.55887 (16)	0.0317 (4)	
C12	0.3203 (2)	0.2616 (2)	0.59846 (19)	0.0488 (6)	
H14	0.3690	0.1939	0.5724	0.059*	
H15	0.3558	0.3211	0.5607	0.059*	
C13	0.1246 (2)	0.53539 (18)	0.67400 (16)	0.0345 (4)	
H16	0.0528	0.4656	0.6217	0.041*	
C14	0.1440 (2)	0.6415 (2)	0.6504 (2)	0.0446 (5)	
H17	0.0860	0.6451	0.5838	0.054*	
C15	0.2493 (2)	0.7428 (2)	0.7254 (2)	0.0459 (5)	
H18	0.2664	0.8168	0.7102	0.055*	
C16	0.3291 (2)	0.73469 (18)	0.82255 (19)	0.0375 (4)	
H19	0.4015	0.8034	0.8757	0.045*	
C17	0.30264 (17)	0.62549 (15)	0.84201 (15)	0.0255 (3)	
C18	0.38677 (18)	0.61428 (15)	0.94787 (16)	0.0282 (3)	
H20	0.4892	0.6238	0.9393	0.034*	
H21	0.3805	0.6801	1.0230	0.034*	
P1	0.59552 (5)	0.02974 (4)	0.74358 (4)	0.03228 (11)	
F4	0.5841 (2)	0.0626 (2)	0.88218 (15)	0.0891 (6)	
F5	0.61092 (18)	0.00030 (18)	0.60798 (13)	0.0725 (5)	
F6	0.76632 (13)	0.04945 (12)	0.77770 (13)	0.0494 (3)	
F7	0.61897 (16)	0.17125 (12)	0.76545 (17)	0.0680 (5)	
F8	0.42616 (14)	0.01136 (14)	0.71068 (15)	0.0597 (4)	
F9	0.57607 (18)	−0.10966 (14)	0.7258 (2)	0.0789 (5)	
F1A	0.2162 (3)	0.4114 (5)	0.3777 (4)	0.0633 (9)	0.60
F2A	0.4293 (6)	0.4201 (7)	0.3364 (5)	0.0959 (18)	0.60
F3A	0.3715 (5)	0.5861 (4)	0.4479 (2)	0.0956 (15)	0.60
F1B	0.2326 (11)	0.3584 (9)	0.3289 (9)	0.075 (3)*	0.24
F2B	0.4556 (11)	0.4729 (9)	0.3644 (10)	0.048 (2)*	0.24
F3B	0.3095 (15)	0.5496 (11)	0.4460 (11)	0.097 (4)*	0.24
F1C	0.3378 (19)	0.3456 (13)	0.2889 (13)	0.091 (4)*	0.16
F2C	0.4376 (13)	0.5252 (12)	0.4082 (13)	0.063 (3)*	0.16
F3C	0.2240 (13)	0.4706 (12)	0.4110 (11)	0.054 (3)*	0.16
O4	0.15453 (15)	0.51056 (17)	0.21720 (16)	0.0494 (4)	
O5	0.38100 (14)	0.53211 (14)	0.18470 (12)	0.0372 (3)	
C19	0.28069 (19)	0.50937 (18)	0.23870 (16)	0.0320 (4)	
C20	0.3227 (3)	0.4746 (3)	0.3470 (2)	0.0605 (7)	

### Atomic displacement parameters (Å^2^)

**Table:**

	*U*^11^	*U*^22^	*U*^33^	*U*^12^	*U*^13^	*U*^23^
Ni1	0.01984 (11)	0.02370 (12)	0.02007 (11)	0.00408 (8)	0.00167 (7)	0.00581 (8)
O1	0.0279 (6)	0.0369 (7)	0.0343 (7)	0.0153 (5)	0.0108 (5)	0.0175 (6)
O2	0.0225 (6)	0.0426 (7)	0.0292 (6)	0.0059 (5)	0.0058 (5)	0.0058 (5)
O3	0.0354 (7)	0.0307 (6)	0.0223 (6)	−0.0020 (5)	−0.0054 (5)	0.0102 (5)
N1	0.0229 (6)	0.0228 (6)	0.0273 (7)	0.0037 (5)	−0.0003 (5)	0.0064 (5)
N2	0.0275 (7)	0.0248 (7)	0.0227 (7)	0.0042 (5)	0.0015 (5)	0.0055 (5)
N3	0.0244 (7)	0.0279 (7)	0.0217 (7)	0.0032 (5)	0.0020 (5)	0.0085 (5)
C1	0.0264 (8)	0.0289 (9)	0.0426 (10)	0.0080 (7)	0.0000 (7)	0.0099 (8)
C2	0.0370 (10)	0.0365 (10)	0.0634 (14)	0.0103 (8)	−0.0038 (9)	0.0243 (10)
C3	0.0543 (13)	0.0514 (13)	0.0652 (15)	0.0095 (10)	0.0025 (11)	0.0402 (12)
C4	0.0453 (12)	0.0497 (12)	0.0522 (13)	0.0087 (9)	0.0137 (10)	0.0313 (11)
C5	0.0263 (8)	0.0252 (8)	0.0304 (8)	0.0019 (6)	0.0023 (6)	0.0085 (7)
C6	0.0247 (8)	0.0284 (8)	0.0344 (9)	0.0035 (6)	0.0079 (7)	0.0105 (7)
C7	0.0280 (8)	0.0312 (9)	0.0293 (9)	0.0016 (7)	−0.0013 (7)	0.0117 (7)
C8	0.0383 (10)	0.0316 (9)	0.0357 (10)	−0.0020 (7)	−0.0101 (8)	0.0131 (8)
C9	0.0574 (13)	0.0314 (9)	0.0267 (9)	0.0082 (9)	−0.0084 (8)	0.0040 (8)
C10	0.0544 (12)	0.0372 (10)	0.0248 (9)	0.0169 (9)	0.0052 (8)	0.0044 (8)
C11	0.0346 (9)	0.0300 (9)	0.0269 (8)	0.0094 (7)	0.0046 (7)	0.0051 (7)
C12	0.0325 (10)	0.0682 (15)	0.0309 (10)	0.0091 (9)	0.0099 (8)	0.0007 (10)
C13	0.0352 (9)	0.0379 (10)	0.0258 (8)	0.0003 (7)	−0.0039 (7)	0.0132 (7)
C14	0.0460 (11)	0.0492 (12)	0.0394 (11)	0.0012 (9)	−0.0067 (9)	0.0269 (10)
C15	0.0483 (12)	0.0403 (11)	0.0511 (13)	0.0000 (9)	−0.0021 (10)	0.0282 (10)
C16	0.0339 (9)	0.0326 (9)	0.0414 (10)	−0.0022 (7)	−0.0023 (8)	0.0159 (8)
C17	0.0210 (7)	0.0298 (8)	0.0247 (8)	0.0049 (6)	0.0050 (6)	0.0092 (7)
C18	0.0257 (8)	0.0256 (8)	0.0283 (8)	0.0018 (6)	−0.0021 (6)	0.0080 (7)
P1	0.0319 (2)	0.0294 (2)	0.0314 (2)	0.00634 (18)	0.00592 (18)	0.00648 (19)
F4	0.0957 (13)	0.1473 (19)	0.0426 (9)	0.0587 (13)	0.0304 (9)	0.0368 (11)
F5	0.0719 (10)	0.1113 (14)	0.0344 (7)	0.0355 (10)	0.0129 (7)	0.0187 (8)
F6	0.0357 (6)	0.0454 (7)	0.0592 (8)	0.0106 (5)	0.0001 (5)	0.0114 (6)
F7	0.0577 (9)	0.0331 (7)	0.1023 (13)	0.0123 (6)	−0.0049 (8)	0.0157 (8)
F8	0.0328 (7)	0.0575 (8)	0.0740 (10)	0.0086 (6)	0.0046 (6)	0.0086 (7)
F9	0.0600 (9)	0.0381 (7)	0.1392 (17)	0.0055 (7)	0.0159 (10)	0.0374 (9)
F1A	0.0651 (18)	0.089 (3)	0.061 (2)	0.0150 (19)	0.0181 (15)	0.059 (2)
F2A	0.082 (3)	0.167 (5)	0.110 (4)	0.085 (4)	0.032 (3)	0.105 (4)
F3A	0.075 (2)	0.159 (4)	0.0224 (12)	−0.018 (3)	−0.0068 (13)	0.0261 (17)
O4	0.0281 (7)	0.0754 (11)	0.0610 (10)	0.0183 (7)	0.0077 (6)	0.0425 (9)
O5	0.0281 (6)	0.0558 (8)	0.0263 (6)	0.0041 (6)	0.0028 (5)	0.0171 (6)
C19	0.0276 (8)	0.0416 (10)	0.0291 (9)	0.0093 (7)	0.0032 (7)	0.0158 (8)
C20	0.0362 (11)	0.114 (2)	0.0521 (14)	0.0214 (13)	0.0112 (10)	0.0533 (16)

### Geometric parameters (Å, °)

**Table:**

Ni1—O1	2.0461 (12)	C10—C11	1.389 (3)
Ni1—N2	2.0601 (14)	C10—H13	0.9500
Ni1—O2	2.0647 (12)	C11—C12	1.506 (3)
Ni1—N1	2.0662 (14)	C12—H14	0.9900
Ni1—O3	2.0714 (12)	C12—H15	0.9900
Ni1—N3	2.0769 (14)	C13—C14	1.374 (3)
O1—C6	1.421 (2)	C13—H16	0.9500
O1—H1	0.8716	C14—C15	1.381 (3)
O2—C12	1.417 (2)	C14—H17	0.9500
O2—H2	0.9235	C15—C16	1.377 (3)
O3—C18	1.422 (2)	C15—H18	0.9500
O3—H3	0.9822	C16—C17	1.384 (3)
N1—C5	1.340 (2)	C16—H19	0.9500
N1—C1	1.352 (2)	C17—C18	1.501 (2)
N2—C11	1.342 (2)	C18—H20	0.9900
N2—C7	1.346 (2)	C18—H21	0.9900
N3—C17	1.340 (2)	P1—F5	1.5697 (15)
N3—C13	1.347 (2)	P1—F9	1.5777 (15)
C1—C2	1.377 (3)	P1—F7	1.5881 (14)
C1—H4	0.9500	P1—F4	1.5915 (16)
C2—C3	1.376 (4)	P1—F8	1.5960 (14)
C2—H5	0.9500	P1—F6	1.6087 (13)
C3—C4	1.385 (3)	F1A—C20	1.308 (4)
C3—H6	0.9500	F2A—C20	1.316 (4)
C4—C5	1.387 (3)	F3A—C20	1.417 (5)
C4—H7	0.9500	F1B—C20	1.420 (10)
C5—C6	1.503 (2)	F2B—C20	1.284 (11)
C6—H8	0.9900	F3B—C20	1.255 (12)
C6—H9	0.9900	F1C—C20	1.505 (14)
C7—C8	1.380 (3)	F2C—C20	1.188 (11)
C7—H10	0.9500	F3C—C20	1.279 (12)
C8—C9	1.381 (3)	O4—C19	1.223 (2)
C8—H11	0.9500	O5—C19	1.249 (2)
C9—C10	1.376 (3)	C19—C20	1.536 (3)
C9—H12	0.9500		
			
O1—Ni1—N2	98.09 (6)	N2—C11—C10	121.78 (18)
O1—Ni1—O2	172.33 (5)	N2—C11—C12	117.35 (16)
O1—Ni1—N1	78.11 (5)	C10—C11—C12	120.87 (18)
N2—Ni1—O2	78.53 (5)	O2—C12—C11	109.50 (16)
N2—Ni1—N1	97.35 (6)	O2—C12—H14	109.8
O2—Ni1—N1	95.41 (6)	C11—C12—H14	109.8
O1—Ni1—O3	95.00 (5)	O2—C12—H15	109.8
N2—Ni1—O3	166.15 (6)	C11—C12—H15	109.8
O2—Ni1—O3	89.01 (5)	H14—C12—H15	108.2
N1—Ni1—O3	89.74 (5)	N3—C13—C14	122.91 (17)
O1—Ni1—N3	93.77 (5)	N3—C13—H16	118.5
N2—Ni1—N3	96.49 (6)	C14—C13—H16	118.5
O2—Ni1—N3	93.45 (6)	C13—C14—C15	118.77 (18)
N1—Ni1—N3	164.81 (6)	C13—C14—H17	120.6
O3—Ni1—N3	78.09 (5)	C15—C14—H17	120.6
C6—O1—Ni1	118.49 (10)	C16—C15—C14	118.86 (18)
C6—O1—H1	112.8	C16—C15—H18	120.6
Ni1—O1—H1	122.2	C14—C15—H18	120.6
C12—O2—Ni1	115.90 (11)	C15—C16—C17	119.37 (18)
C12—O2—H2	114.4	C15—C16—H19	120.3
Ni1—O2—H2	118.1	C17—C16—H19	120.3
C18—O3—Ni1	117.12 (10)	N3—C17—C16	122.05 (16)
C18—O3—H3	106.7	N3—C17—C18	117.36 (15)
Ni1—O3—H3	130.7	C16—C17—C18	120.58 (15)
C5—N1—C1	118.61 (16)	O3—C18—C17	110.11 (13)
C5—N1—Ni1	116.13 (11)	O3—C18—H20	109.6
C1—N1—Ni1	124.57 (13)	C17—C18—H20	109.6
C11—N2—C7	118.69 (15)	O3—C18—H21	109.6
C11—N2—Ni1	115.61 (12)	C17—C18—H21	109.6
C7—N2—Ni1	125.69 (12)	H20—C18—H21	108.2
C17—N3—C13	118.01 (15)	F5—P1—F9	90.77 (11)
C17—N3—Ni1	116.35 (11)	F5—P1—F7	90.50 (11)
C13—N3—Ni1	125.64 (12)	F9—P1—F7	178.09 (10)
N1—C1—C2	122.16 (19)	F5—P1—F4	178.33 (12)
N1—C1—H4	118.9	F9—P1—F4	90.26 (12)
C2—C1—H4	118.9	F7—P1—F4	88.43 (12)
C3—C2—C1	119.20 (19)	F5—P1—F8	90.97 (9)
C3—C2—H5	120.4	F9—P1—F8	91.58 (9)
C1—C2—H5	120.4	F7—P1—F8	89.83 (8)
C2—C3—C4	119.1 (2)	F4—P1—F8	90.31 (10)
C2—C3—H6	120.5	F5—P1—F6	89.37 (8)
C4—C3—H6	120.5	F9—P1—F6	88.81 (8)
C3—C4—C5	119.1 (2)	F7—P1—F6	89.77 (7)
C3—C4—H7	120.5	F4—P1—F6	89.35 (9)
C5—C4—H7	120.5	F8—P1—F6	179.48 (9)
N1—C5—C4	121.87 (17)	O4—C19—O5	129.17 (18)
N1—C5—C6	117.09 (15)	O4—C19—C20	115.85 (17)
C4—C5—C6	121.03 (17)	O5—C19—C20	114.97 (16)
O1—C6—C5	108.67 (14)	F2C—C20—F3C	111.5 (9)
O1—C6—H8	110.0	F3B—C20—F2B	102.8 (7)
C5—C6—H8	110.0	F1A—C20—F2A	109.8 (4)
O1—C6—H9	110.0	F1A—C20—F3A	104.4 (3)
C5—C6—H9	110.0	F2A—C20—F3A	106.6 (4)
H8—C6—H9	108.3	F3B—C20—F1B	108.1 (7)
N2—C7—C8	122.40 (18)	F2B—C20—F1B	110.5 (6)
N2—C7—H10	118.8	F2C—C20—F1C	102.0 (9)
C8—C7—H10	118.8	F3C—C20—F1C	107.2 (8)
C7—C8—C9	118.65 (18)	F2C—C20—C19	118.3 (6)
C7—C8—H11	120.7	F3B—C20—C19	113.8 (6)
C9—C8—H11	120.7	F3C—C20—C19	113.1 (6)
C10—C9—C8	119.43 (18)	F2B—C20—C19	113.3 (5)
C10—C9—H12	120.3	F1A—C20—C19	115.0 (2)
C8—C9—H12	120.3	F2A—C20—C19	113.6 (3)
C9—C10—C11	119.03 (19)	F3A—C20—C19	106.5 (3)
C9—C10—H13	120.5	F1B—C20—C19	108.2 (4)
C11—C10—H13	120.5	F1C—C20—C19	103.1 (6)

### Hydrogen-bond geometry (Å, °)

**Table:**

*D*—H···*A*	*D*—H	H···*A*	*D*···*A*	*D*—H···*A*
O1—H1···O4^i^	0.87	1.76	2.6003 (19)	162.5
O2—H2···O5^ii^	0.92	1.77	2.6965 (18)	175.8
O3—H3···O5^iii^	0.98	1.65	2.6267 (18)	173.8

Symmetry codes: (i) −*x*, −*y*+1, −*z*+1; (ii) −*x*+1, −*y*+1, −*z*+1; (iii) *x*, *y*, *z*+1.
